# Impact of Cavitation Jet on the Structural, Emulsifying Features and Interfacial Features of Soluble Soybean Protein Oxidized Aggregates

**DOI:** 10.3390/foods12050909

**Published:** 2023-02-21

**Authors:** Yanan Guo, Caihua Liu, Yichang Wang, Shuanghe Ren, Xueting Zheng, Jiayu Zhang, Tianfu Cheng, Zengwang Guo, Zhongjiang Wang

**Affiliations:** College of Food Science, Northeast Agricultural University, Harbin 150030, China

**Keywords:** soybean protein, soluble oxidized aggregates, emulsifying properties, rheological properties, cavitating jet

## Abstract

A cavitation jet can enhance food proteins’ functionalities by regulating solvable oxidized soybean protein accumulates (SOSPI). We investigated the impacts of cavitation jet treatment on the emulsifying, structural and interfacial features of soluble soybean protein oxidation accumulate. Findings have shown that radicals in an oxidative environment not only induce proteins to form insoluble oxidative aggregates with a large particle size and high molecular weight, but also attack the protein side chains to form soluble small molecular weight protein aggregates. Emulsion prepared by SOSPI shows worse interface properties than OSPI. A cavitation jet at a short treating time (<6 min) has been shown to break the core aggregation skeleton of soybean protein insoluble aggregates, and insoluble aggregates into soluble aggregates resulting in an increase of emulsion activity (EAI) and constancy (ESI), and a decrease of interfacial tension from 25.15 to 20.19 mN/m. However, a cavitation jet at a long treating time (>6 min) would cause soluble oxidized aggregates to reaggregate through an anti-parallel intermolecular β-sheet, which resulted in lower EAI and ESI, and a higher interfacial tension (22.44 mN/m). The results showed that suitable cavitation jet treatment could adjust the structural and functional features of SOSPI by targeted regulated transformation between the soluble and insoluble components.

## 1. Introduction

Soybean is an important crop with seeds that contain abundant protein of approximately 40% [1]. The soybean protein has different physiological impacts including dropping blood lipids, blood pressure, and inhibiting cardiovascular and cerebrovascular disease indirectly [2]. Therefore, soybean proteins have been extensively exploited in food and feed plants due to their superior nutritious rate, high functional features, and low price [3]. Studies have revealed that in 2019, global soy production reached 366.67 million tons [4], which caused huge storage and transportation pressures. In addition, soy protein is vulnerable to oxidative attack during storage and transportation. The parties within the molecule re-syndicate to create oligomers following disclosure, owing to the oxidative denaturation of soy protein, which further forms macromolecular aggregates due to hydrophobicity and electrostatic attraction [5]. It is challenging to use oxidized soy protein in food manufacturing, because the formation of insoluble aggregates in protein aggregates is a significant factor in the loss of some biological and functional features of proteins, for instance, protein solubility, emulsifying effects, and emulsifying stability [6].

The physical control of oxidized protein aggregates is currently the subject of extensive research. The degree of whey protein isolate (WPI) aggregation’s cross-linking could be controlled, and its gel and emulsifying characteristics could be improved, expanding the use of WPI in food processing, according to [7]. A decrease in the aggregate concentration of β-conglycinin and a rise in the size of the solvable accumulates for glycinin and soy protein insulate were found by Keerati-U-Rai et al. (2009) [8], who also established that dynamic extra-pressure homogenization triggered a transformation from insoluble aggregate to soluble aggregate. Additionally, Cao et al. (2021) [9] discovered that using ultrasound might modify the intermolecular interactions, alter the shape and accumulation of oxidized quinoa proteins, and increase the quantity of soluble aggregates, refining the functional attributes of the quinoa proteins. In a study by Zhang (2020) [10], it was demonstrated that ultrasonic treatment could prevent casein molecules from self-aggregating in a solution, as well as deteriorate the accumulation brought on by interfacial adsorption through foam fractional process, resulting in an improved protein aggregate function. Physical fields can therefore cause subunit dissociation and aggregation to directly regulate the protein structure, which eventually results in an improvement in the functional characteristics of protein aggregates. However, because of their high power requirements and limited effort capabilities in food processing procedures, high-pressure homogenization and ultrasonic processes were unable to be extensively utilized.

A cavitation jet is a water jet that can produce the cavitation effect; it can induce the rapid vaporization of the liquid to form many cavitation bubbles. After the liquid flows into the high-pressure zone, these cavitation bubbles will collapse and extinguish, resulting in the generation of an extra-velocity turbulent shear and a substantial pressure differential and molecular impact, which could cause big particles to break up into smaller ones and the structure of food to be refined, which will affect its functional characteristics [11]. The cavitation jet technique provides benefits over alternative mechanical treatments, including ease of use, speedy processing, low processing temperatures, and cheap processing costs [12]. Thus, in the realm of food processing, the cavitation jet treatment may be employed as an effective and energy-saving processing method. Cavitating jets could alter the structure and characteristics of proteins, as well as eliminate the hydrophobic and electrostatic connections between molecules. Based on this, researchers have shown that cavitation treatments may change the structures of the protein isolate and promote emulsifying characterizations [13]. The previous research results revealed that the appropriate time of cavitation jet treating could damage the structure of protein-oxidized accumulates and improve the emulsification and interface properties. In addition, this outcome might be associated with the regulation of the cavitation jet on the oxidized masses and the induction of conversion between the soluble and insoluble oxidized aggregates. Nevertheless, in the current work, the research on the transformation law between the soluble and insoluble components of protein oxidative aggregates was less. It is limited by the tender of the cavitation jet physical field in the governing of the protein accumulates and the analysis of its mechanism.

Thus, in this study, soybean protein was utilized as the investigation entity, and 2,2′-azobis(2-amidinopropane) dihydrochloride (AAPH) was employed to create an oxidation aggregation system of soy protein. By mimicking the definite creation of oxidized protein accumulates in the means of factory storing, the oxidized protein accumulates were treated with several cavitation jet times (0–15 min), and the soluble aggregates with the cavitation jet treatment were obtained during the centrifugation. We studied the change of the structure and the emulsifying and interfacial descriptions of the soluble component in protein accumulates after cavitation jet treatment. The mechanisms of the cavitation jet governing the oxidative accumulates of soybean protein and transformation law between soluble and insoluble components were explained at the molecular level. This might cause enhancements in the function of the cavitation jet in the soy protein plant, and deliver a theoretic base for the claim of the development, alterations, storing, and shipping of the soybean protein stuffs.

## 2. Materials and Methods

### 2.1. Materials

Shandong Yuwang ecological food industry Plant Limited provided the soy protein isolate (92.4% protein) (Shandong, China). From Beijing Dingguo Changsheng Biotechnology Co. Ltd., chemicals such as 2,2′-dithiobis(5-nitropyridine) (DTNP) and 8-Anilino-1-Naphthalene Sulfonate (ANS) were acquired (Beijing, China).

### 2.2. Formulation of Soluble Soybean Protein Oxidized Accumulates

According to a prior work, the soluble oxidized accumulates of soybean protein (SOSPI) were produced [14]. To generate a 10 mg mL^−1^ soybean protein mix, the soybean protein was liquified in a phosphate buffer solution (PBS) with a phosphate dose of 0.01 mol L^−1^, a pH of 7.2, and 0.5 mg mL^−1^ of NaN_3_. The final concentration of AAPH was increased to 0.5 mmol L^−1^ by adding AAPH. The soybean protein oxidized accumulate mix with a 12 h oxidation period was prepared after oxidating treatment for 12 h at 37 °C and became murky. Dialysis was performed at 4 °C for 72 h using a 14,000 kDa dialysis bag, and the deionized water (dH_2_O) was replaced every 6 h. The samples were gathered and given the designation OSPI after spray drying. The soybean protein oxidized accumulate mix underwent a 12 h oxidation process before standing centrifugated at 4 °C for 20 min at a rapidity of 9000 rpm to discrete the soluble components from the insoluble components. The samples were gathered and given the name SOSPI after spray drying.

### 2.3. Formulation of Samples for Cavitation Jet Treatment

The 2 L OSPI (25 °C, 10 mg mL^−1^) was poured into the SL-2 cavitation jet machine (Zhongsen Huijia Technology Development Co., Ltd., Beijing, China) to treat at 80 MPa for six diverse times: 2, 4, 6, 8, 10, and 15 min. The SOSPI was liquified in 0.01 mol/L PBS (pH 7.2, comprising 0.5 mg mL^−1^ NaN_3_). After treatment, the protein was immediately chilled in an ice bath for 15 min, and trailed by 20 h of centrifugal treatment at 4 °C and 9000 rpm to remove the insoluble parts. Spray drying was used to create all sample solutions, which were given the names SCOSPI-2 min, SCOSPI-4 min, SCOSPI-6 min, SCOSPI-8 min, SCOSPI-10 min, and SCOSPI-15 min. Three groups of parallel samples were taken.

### 2.4. Measuring the Particle Size Dispersal

Based on the technique labeled by Ma et al. (2019), the particle size dispersal (PSD) was estimated via a laser scattering Mastersizer S (Malvern, UK) and a 300 inverse Fourier lens with the relief of a He–Ne laser λ = 633 [15]. The protein’s refractive index was 1.33 when the amount was made at room temperature (RT, 25 ± 2 °C). Before measurement, the samples were diluted with dH_2_O to 50 mg/mL, and the particle sizes ranged between 0–10,000 μm.

### 2.5. Measurement of the Molecular Weight Circulation

Following Ma et al. (2019) [15], examples of soybean protein were examined using an HPLC unit (Milford, MA, USA). Briefly, the molecular weight of the proteins at 280 nm was determined via a Waters 2175 UV finder (Milford, MA, USA).

### 2.6. Measurement of the Fourier Transform Infrared Spectroscopy (FTIR) Spectroscopy

A Bruker Vertex 70 was used to analyze the materials using Fourier transform infrared (FTIR) (Bruker Optics GmbH, Ettlingen, Germany). At 0.5 cm^−1^ tenacity and RT (25 ± 2 °C), a total of 64 scans were found between 4000 and 400 cm^−1^. The secondary structure was determined using the FTIR spectra’s secondary-derivation and deconvolution processes, and it was based on the amide I band (1600–1700 cm^−1^). According to Tang et al. (2009), the method involved the secondary structure of the proteins being examined using Peakfit Ver., 4.12 software, and the algorithm utilized was Gaussian peak fitting [16].

### 2.7. Measuring of the Fluorescence Emission Spectra

According to the technique used by Jiang et al. (2014), the fluorescence emission spectra of the materials were found via a Hitachi F-7000 fluorescence spectrophotometer (Hitachi Inc., Tokyo, Japan) [17]. The soybean protein trials were thinned in 0.01 mol L^−1^ PBS to a protein dose of 0.2 mg mL^−1^ to produce emission spectra at an excitation wavelength of 295 nm and from 300 to 400 nm. By employing a fixed 5 nm for both the emission and excitation in triplicate, the bandwidths were attained.

### 2.8. Measurement of the Sulfhydryl Content

According to the Wu et al. (2019) approach, the amounts of disulfide bonds and free sulfhydryl (SH) assemblies were measured [18]. DTNP was used in a variation of Ellman’s approach to ascertain the SH cluster insides in the trial. The molar extinction constant (13,600 M^−1^ cm^−1^) was utilized to represent the SH contents as a nmol mg^−1^ protein.

### 2.9. Measuring of the Transmission Electron Microscopy (TEM)

TEM was dedicated by utilizing a previously described technique [19]. After being diluted 350 times in dH2O, the sample was dispensed in 30 μL droplets and applied on a carbon net (200 mesh). The surplus was wiped away using permeable paper after 120 s. The net was air-dehydrated on sieve paper after the samples were dyed for 3 min with a 2% uranyl acetate solution. Benefitting a TEM-JEM-1230 (JEOL, Tokyo, Japan) with a hastening voltage of 80 kV, the morphology of the sample was examined.

### 2.10. Measuring of the Emulsifying Activity Index (EAI) and Emulsion Solidity Index (ESI)

The Kevin et al. (1978) approach was used to evaluate the EAI and ESI [20]. A high-rapidity homogenizer (T-25 homogenizer, IKA, Staufen, Germany) was used to combine a 15 mL sample of a 0.1% (*w*/*v*) protein mix with 5 mL of maize oil at 7200× *g* for 10 min to create an emulsion. The emulsion was then detached from the lowest of the centrifuge tubes and normalized for 0 and 30 min before being diluted 100 times with 5 mL 0.1% sodium dodecyl sulfate. A spectrophotometer was used to test the absorbance at 500 nanometers (Beckman DU 500, Fullerton, CA, USA). The EAI and ESI were stated as:

$$\mathrm{EAI}(m^{2}/g)=\frac{2\times 2.303\times \mathrm{DF}\times A_{0}}{(1-\theta )\times C\times \phi \times 10000}\;\mathrm{ESI}(\%)=\frac{A_{0}}{A_{0}-A_{30}}\times 10$$

where A_0_ is the absorbance at 0 min of the thinned emulsion, DF is the dilution aspect (×100), c is the model dose (g mL^−1^), ϕ is the pictorial path, θ is the portion of the oil (0.25), and A_30_ is the absorbance after 30 min.

### 2.11. Measurement of the Confocal Laser Scanning Microscope (CLSM)

The Leica TCS SP2 CLSM was used to study the microstructure of emulsions. To create an emulsion, 15 mL of a 0.1% (*w*/*v*) protein mix was normalized with 5 mL of maize oil at 7200× *g* for 30 min. A 1 mL of emulsion was added to the dye (40 μL), which included 0.02% Nile red dye and 0.1% Nile blue dye. After that, a coverslip was put on top of the colored emulsion in the middle of the slide. To prevent the water from evaporating, silicone oil was sprayed to the superiority of the coverslip. The emphasis plane was originally changed following an inspection with a 100× impartial lens, while the slide was mounted on a laser confocal microscope phase. Pre-examining was performed with Ar ion at 488 nm and a He/Ne ion laser at 633 nm. A fluorescence figure was composed with a visualizing intensity of 1024 × 1024.

### 2.12. Measuring of the Quantity of Adsorbed Proteins at Interface (AP%)

According to Liang and Tang, the amount of adsorbed proteins at the interface (AP%) of these emulsion samples was calculated [21]. A 10,000 g centrifuge was used to spin each new emulsion (1 mL) for 45 min at RT. A cream coat (or concerted oil droplets) at the upper of the tube and the aqueous stage of the emulsion at the bottom were visible after centrifugation. A 0.22 μm filter was utilized to sieve the supernatant after the cream layer was delicately detached using a syringe (Millipore Corp.). The Lowry technique was utilized to estimate the filtrate’s protein content, with a BSA serving as the reference. To estimate the protein intensity (*C_s_*) in the upper phase, the initial protein mix was likewise centrifuged under identical circumstances. The *AP (%)* was expressed as:

$$AP\;\left(\%\right)=\frac{C_{S}-C_{f}}{C_{0}}\times 100$$

where *C_s_* is the content of preliminary protein solution in the supernatant (mg), *C_f_* is the content of protein in filtrate after centrifugation (mg), and *C*_0_ is the preliminary protein intensity of the protein mixes concerned for the emulsion formulation (mg).

### 2.13. Measurement of the Interfacial Tension

Various materials’ surface tension was estimated via an automated surface tensiometer (DCAT21, Data Physics Instruments GmbH, Filderstadt, Germany). A total of 20 mL of the sample mix was then put into a 25 mL cylinder after the protein model had first been dissolved in dH_2_O (1%, *m*/*v*). The apparatus’s measuring variety was always between 1 and 100 mN m^−1^, with a SD that never went beyond 0.03 mN m^−1^.

### 2.14. Measurement of the Viscoelastic Properties

The Sun et al. (2012) approach is used to assess the viscoelastic characteristics of emulsions [22]. An RST-CPS rheometer was used to measure the sample emulsions’ rheological characteristics (Brookfield, Middleboro, MA, USA). At a temperature of 40 °C, the samples were sandwiched between two parallel plates with 1 mm space among them. A strain examining the analysis performed at an incidence of 1 Hz was used to identify the linear viscoelastic area of each sample. Each protein sample’s elastic and storage moduli were determined in the linear viscoelastic area.

### 2.15. Measurement of the Apparent Viscosity

Rendering to the technique delineated by Swa et al. (2020), rheological tests were carried out via an AR 1500 regulated stress rheometer (TA, West Sussex, UK) outfitted with cone and bowl geometries (40 mm, angle 1°, and gap 0.100 mm) [23]. The same technique was used to create the sample emulsions. The sample emulsions were divided into 2.0 mL aliquots and placed on the stage for measurement at 25 ± 0.1 °C. After 5 min, the viscosity ranged from 0 to 200 s^−1^. Using the program, the measuring was performed in triplicate. We matched the investigational flow curves to Sisko’s pattern that provided the finest fit and was signified by:

$$\eta =\eta_{0}+K\gamma^{n-1}$$

where *η* is the ostensible viscosity (Pa·s), *η*_0_ is the vintage ostensible viscosity (Pa·s), *K* is the consistency index (Pa·s^n^), *γ* is the shear ratio (s^−1^), and *n* is the performance index (dimensionless).

### 2.16. Statistical Analysis

Statistical assessment was accomplished via SPSS ver. 20.0. The outcomes were imperiled to Duncan’s multiple series and ANOVA tests. All the rates gained are stated as the mean ± SD in triplicate. A *p*-value ≤ 0.05 was measured significantly.

## 3. Results

### 3.1. Particle Size Distribution and Molecular Weight Circulation

**Figure 1:**
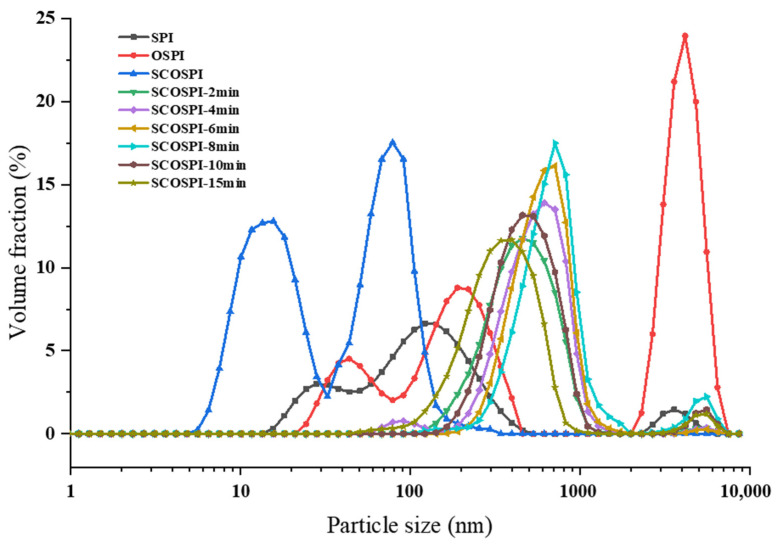
Particle size distribution (PSD) of natural, oxidized soybean protein, and the cavitation jet treated on soluble soybean protein oxidized accumulates at several times (2, 4, 6, 8, 10, and 15 min).

**Figure 2:**
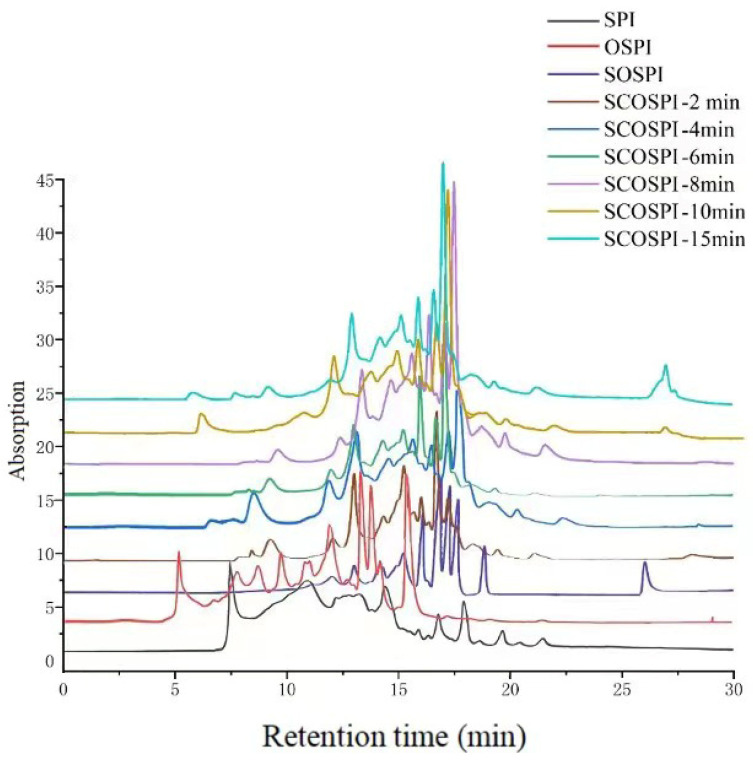
SEC profiles of natural, oxidized soybean protein, and cavitation jet treated on soluble soybean protein oxidized accumulates at several times (2, 4, 6, 8, 10, and 15 min).

The SEC-HPLC and particle size dispersal can characterize the molecular weight, size, and aggregation degree of the soluble components in soybean protein oxidized aggregates treated by cavitation jet. It can be seen from Figure 1 and Figure 2 and Table 1 that, equated with SPI, the particle size of OSPI showed a unimodal particle size and lifted to the right, meaning the average particle size increased significantly. Furthermore, the elution time of the first molecular weight peak of OSPI diminished and the peak quantity increased. However, as a soluble component in OSPI, the particle size of SOSPI displayed a bimodal particle size, the initial particle size peak transferred to the left, and the average particle size decreased. The elution time of the first molecular weight peak of SOSPI increased and the peak area decreased. The results revealed that after the oxidation treatment, the oxidized accumulates with a huge particle size and a high molecular weight were insoluble aggregates, and the soluble components were proteins with a small particle size and a low molecular weight. Radicals in the oxidative environment could induce proteins to form insoluble oxidative aggregates through covalent crosslinking, but they will also attack protein side chains to form small molecular weight soluble proteins [24].

With the increase of the cavitation jet treatment time, the retention time of the initial elution peak and the peak area of the protein components with a small molecular weight of SCOSPI decreased, and the particle size peak of SCOSPI lifted to the right. When the cavitation jet treatment time was 8 min, the first particle size peak of SCOSPI moved to the maximum right, and the average particle size achieved the highest. The outcomes displayed that the molecular weight and particle size of SCOSPI with the cavitation jet treatment increased, and the low molecular weight and small particle size protein components declined. The cavitation jet treatment could promote the depolymerization of insoluble aggregates in OSPI and transform them into soluble oxidized aggregates through high shear and cavitation effects, developing an increase of the particle size and molecular weight of the soluble oxidized accumulates [8]. Moreover, the cavitation jet would intensify the collision between the small molecule soluble aggregates, and then polymerize into a bigger particle size and molecular weight soluble protein molecule, resulting in the reduction of small molecular weight protein components [16,25]. When the cavitation jet treatment time exceeded 8 min, the first particle size peak of SCOSPI moved to the left and the retaining time of the initial elution peak and the peak area of protein substances with small molecular weight of SCOSPI amplified, indicating that when the treatment time was too long, the protein molecular weight of SCOSPI decreased and the small molecular weight protein component increased. The thermal effect and free radical effect of the cavitation jet, on the one hand, could promote the further aggregation among proteins to form insoluble aggregates, which were removed by centrifugation. On the other hand, it would split some peptide chains, resulting in soluble protein components dominated by small molecular weight and particle size protein molecules [16,26]. Combined with the research of the team in the early stage [27], cavitation jets can break losing the disulfide bonds and protein skeleton structures that declined the amassed sizes and molecular weights of oxidized aggregates. However, how these components of protein aggregates mutually transform is unclear. Through the particle size and molecular weight of this research, we can obtain that a cavitation jet can also induce the insoluble aggregates to break down under high shear stress and transform into soluble aggregates, ensuing in the rise of the particle size and molecular weight of the solvable accumulates. Consequently, a suitable cavitation jet treatment could adjust the structural and functional attributes of OSPI by inducing the cleavage of insoluble oxidized aggregates and transforming them into soluble aggregate components.

### 3.2. FTIR Spectroscopy

**Figure 3:**
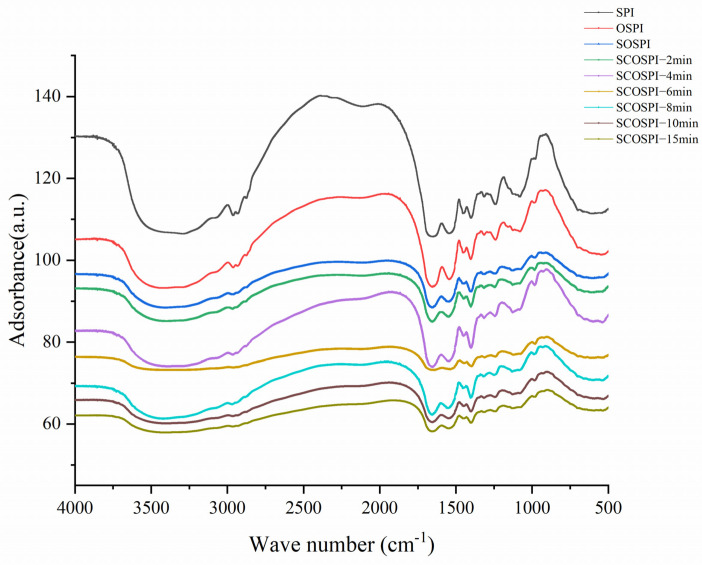
FTIR spectra of natural, oxidized soybean protein, and cavitation jet treated on soluble soybean protein oxidized accumulates at several times (2, 4, 6, 8, 10, and 15 min).

Fourier transform infrared spectroscopy can be utilized to elucidate the secondary structure change of proteins during aggregation and disaggregation [28]. Figure 3 is the FTIR spectra, and Table 2 is the secondary structure of oxidized accumulates and soluble oxidized aggregates after the cavitation jet treatment. Oxidized treatment raised the compounds of β1, β-turn, and γ-random coil in the OSPI and declined the compounds of α-helix. Compared with OSPI, the components of β1 and γ-random coil in SOSPI declined, and the compounds of α-helix increased. α-helix has structured secondary structures featured by high inflexibility and recurrence structure, while γ-random coil has unordered secondary structures featured by plasticity and the deficiency of a recurrence structure [29]. The marker structure of aggregation (β1) is created by molecular interactions during protein oxidation [30]. Changes in the constancy of the H-bond between the amino parties and the polypeptide chain’s carbonyl parties are primarily responsible for the changes in the amount of α-helices [31]. Since the hydrogen connection among the amino and carbonyl groups in the polypeptide chain is unstable, oxidation may attack the amino acid residues in the primary peptide chain, reducing the amount of α-helix present. The spatial structure of a protein heavily influences its functional activities, and proteins with a suitably organized and compact structure exhibit beneficial functional behaviors [32]. Compared with other samples, the lowest α-helix content of OSPI referred that excessive oxidation would seriously demolish the ordered structure of protein. This might be one of the important reasons for the decline of OSPI functional activity [33]. Comparing the results of OSPI and SOSPI, we can find that oxidized protein with several β1 existed in OSPI, while SOSPI has more rigid and ordered structures.

With the increase of the cavitation jet treatment time, β1 of SCOSPI increased first, then decreased and then increased, and other structures showed no obvious regular change trend. Combined with the outcomes of particle size and molecular weight, the superior pressure and superior shear strengths of the cavitation jet at a fleeting treatment time could lead to the cleavage of protein accumulates by weakening the protein–protein interactions and induce insoluble aggregates with high contents of β1 to transform into soluble aggregates resulting in the increase of the β1 contents [34]. Nevertheless, after the treatment time of the cavitation jet exceeded 6 min, the components of β1 of SCOSPI decreased first and then increased. The cavitation jet with long treatment time could induce the soluble aggregate in OSPI to aggregate further, due to the thermal impact and extra-speed instability and formed the insoluble aggregates with high β1 components which were centrifuged and removed, resulting in the decrease of the β1 content. When the cavitation jet treatment time was 8–15 min, the β1 content of SCOSPI increased. Combined with the previous research results of the team [27] during this timeframe, the β1 of the protein oxidized accumulates and soluble aggregates both increased, which showed that continuously extreme cavitation jet treatment can cause the formation of more β1 structures with the aggregation characteristics of soluble and insoluble components. Integrated with the particle size and molecular weight findings, we could find that the particle size and molecular weight of SCOSPI decreased with a long cavitation jet treatment time. This showed that the cavitation jet could depolymerize the soluble components and at the same time could induce the aggregation reaction between protein molecules, resulting in more β1 structures. The above results showed that the control of cavitating jet is an extremely complex process. The cavitation jet might dynamically govern the depolymerization and reaggregation of soluble soybean protein oxidized accumulates through the transformation of the protein spatial structure.

### 3.3. Fluorescence Emission Spectra

**Figure 4:**
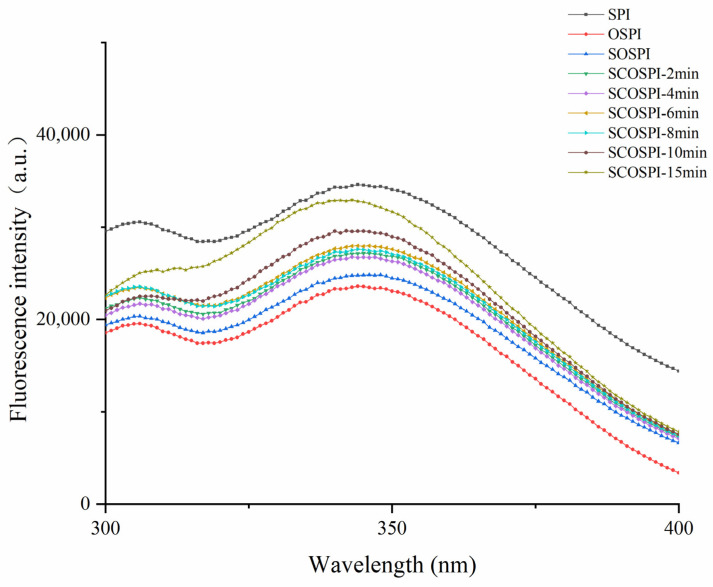
Fluorescence emission spectra of natural, oxidized soybean protein, and cavitation jet treated on soluble soybean protein oxidized accumulates at several times (2, 4, 6, 8, 10, and 15 min).

Fluorescence spectra can characterize the polarity changes of aromatic amino acids in the microenvironment, so as to predict alterations in the tertiary structure of soluble protein aggregates [35]. Figure 4 is the intrinsic fluorescence spectra of the soluble soybean protein oxidized aggregates. Compared with SPI, the fluorescence intensity of OSPI declined significantly and λmax was blue shifted. Compared with OSPI, the fluorescence intensity of SOSPI raised and λmax was red shifted. Free radicals in the oxidizing situation could induce the crosslinking, condensation, and nucleation of SPI, and then form the protein aggregation with a tighter structure [36]. Comparing the fluorescence spectrum of OSPI and SOSPI, we could find that the components with tighter structures in the OSPI components exist in insoluble oxidized aggregates. On the other hand, this showed that the change of the structural tightness degree could reflect the conformational change law of the transformation from a soluble protein to an insoluble protein. With the prolongation of the cavitation jet treatment time, SCOSPI λmax initially raised and then declined, and achieved the supreme when the treatment time was 6 min. This showed that the cavitation jet could alter the spatial structure of SCOSPI to regulate the functional activity. The cavitation jet could cleave and break some insoluble soybean protein oxidation aggregates and induce the creation of soluble oxidation accumulates with a loose structure and a larger particle size. This could cause the increase of the number and exposed degree of aromatic amino acid elements of SCOSPI; to show polar solute circumstances, a red move of λmax of SCOSPI was noted. However, with the further extension of the treatment time, some soluble oxidized aggregates of the soybean protein regrouped and transformed into insoluble aggregates, which were centrifuged, resulting in the reduction of the number of aromatic amino acid elements of SCOSPI [37]. In addition, the other soluble aggregates could form β1 structures and aggregate through covalent cross-linking and hydrophobic interaction in the collision, so that the aromatic amino acids of SCOSPI were buried in the structure [38]. Consequently, excessive cavitation jet treatment will, through this dual effect of aggregation and depolymerization together, cause the λmax of SCOSPI blue shift.

### 3.4. Sulfhydryl Content

SH/SS replacement reactions play a key role in protein accumulation, which can reproduce the impact of physical fields on the depolymerization mechanisms of protein. As shown in Table 3, the free sulfhydryl and total sulfhydryl quantity of OSPI decreased, and the disulfide bond quantity increased compared with SPI. However, compared with OSPI, the free sulfhydryl content of SOSPI rose, and the disulfide bond content decreased. The oxidation treatment could promote the creation of disulfide bonds via the disulfide/sulfhydryl switch reaction. More disulfide bonds reflect the tighter spatial structure of soybean protein, so that the oxidation increased the tightness of the soybean protein molecular space structure [39]. This showed that oxidation could adjust the compactness of the protein structure by changing the disulfide bond, thus affecting its functional activity [40]. In addition, the total sulfhydryl content was also decreasing, and the decay of free sulfhydryl quantity was higher than the raise of disulfide bond content, signifying that oxidation also had a non-reversible oxidation reaction on the soybean protein, inducing the transformation of free sulfhydryl into sulfur compounds without a disulfide bond [41]. For soluble components in OSPI, namely SOSPI, the disulfide bond quantity decreased. Combined with the results of the particle size and fluorescence emission spectra, the oxidized treatment diminished the particle size and amplified the fluorescence intensity of SOSPI, indicating that the oxidized masses of soybean protein after the oxidation treatment were mainly insoluble aggregates, and most of the soluble components show a small particle size and a loose and unfolded structure [40]. With the addition of the cavitation jet treatment time, the contents of free sulfhydryl, total sulfhydryl, and disulfide bonds of SCOSPI amplified first and then declined, but the processing time, corresponding to a maximum value of the three, is inconsistent. This is because there was more than one conversion reaction between free sulfhydryl and disulfide bonds in the system. The cavitation jet treatment could break the core aggregation skeleton of SCOSPI, destroy the intermolecular force and spatial structure, and induce the conversion of disulfide bonds into free sulfhydryl groups, resulting in the increase of the free sulfhydryl content of the soluble oxidized aggregates. At the identical time, combining with the increase particle size findings of SOSPI after the cavitation jet, cavitation could also promote the transformation from insoluble aggregates with high disulfide bonds content into soluble aggregates, so the number of soluble aggregates increased [42], which instigated the expansion of disulfide bond content and free sulfhydryl content of soluble oxidized aggregates. Nevertheless, when the cavitation jet treatment time was too long, high intensity, long-time cavitation, turbulence, and thermal effects would cause the re-aggregation of soluble aggregates and also cause the cracking of all accumulates, which was an irreversible denaturation for protein [43]. To be more specific, when the treatment time was maximized, the cavitation jet would destroy the spatial structure and intermolecular force of the oxidized aggregates, resulting in the reduction of the disulfide bond content, which is well matched with the finding of a decreased particle size. However, the free sulfhydryl groups, formed by the disulfide bond breaking in OSPI, would aggregate with each other to form SCOSPI with a tighter spatial structure, ending in a reduction in the quantity of free sulfhydryl groups. This is matched with the findings of FTIR spectroscopy and fluorescence emission spectra. At the same time, cavitation jet also induced the irreversible reaction of protein sulfhydryl groupings to generate the sulfur-comprising components with non-disulfide bonds. Moreover, the conversion of soluble oxidized aggregates to insoluble oxidized aggregates will also lead to the fluctuation of the disulfide bond and free sulfhydryl quantity. These factors together caused the reduction of free sulfhydryl and disulfide bond quantity. The inconsistent processing time, corresponding to the maximum value of the free sulfhydryl, total sulfhydryl, and disulfide bond, showed that the process of aggregation and depolymerization and the conversion between soluble and non-soluble was a very complex process and needs further research.

### 3.5. Transmission Electron Microscopy (TEM)

**Figure 5:**
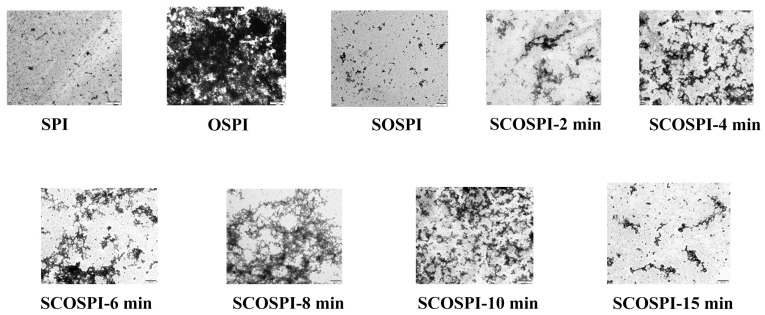
Backbone structure of natural, oxidized soybean protein, and cavitation jet treated on soluble soybean protein oxidized accumulates at several times (2, 4, 6, 8, 10, and 15 min).

To better understand SPI and compare the differences among SPI, OSPI, SOSPI, and SCOSPI, the apparent morphology was visualized by TEM, as shown in Figure 5. Compared with SPI, the aggregation degree of OSPI was increased, and OSPI formed a dense network structure with intense central part. The skeleton structure of SOSPI mainly presented short and small wormlike structures. Oxidation led to the conformational changes of SPI and exposed the side chain groups of hydrophobic aliphatic and aromatic amino acids entrenched within, inducing cross-linking aggregation through hydrophobic interaction. Furthermore, they can also attack the sulfhydryl groups of proteins and convert them into disulfide bonds, showing insoluble protein accumulates with a large particle size and highly cross-linked clusters in OSPI [44]. However, SOSPI showed short rod protein molecules with a small particle size; this is because the proteins with a high degree of cross-linking were transformed into insoluble aggregates and removed by centrifugation [25], as the soluble components of the protein are mainly in the shape of short and small rods. With the increase of the cavitation jet treatment time from 2 min to 8 min, the aggregation degree of SCOSPI increased. Most of the protein aggregates heavily bonded, which consisted of agglomerated smaller worm-like particles, and the skeleton structure became larger and more branches appeared. This is well matched with the outcomes of the particle size and molecular weight. On the one hand, it might be because the cavitation jet broke the disulfide bond of the aggregates, and the insoluble aggregation with large, clustered morphology, resembling those of compact reticulation, was cracked. Then, the insoluble aggregation transformed into soluble aggregates, which led to the amplification of the number of soluble aggregates in the supernatant and presented a cluster structure. On the other hand, it might be that under the cavitation treatment, the fragmentation of the skeleton structure increased. This result promoted the mutual collision between soluble protein molecules and the binding probability of free sulfhydryl clusters, resulting in the enlargement and additional branches of the originally short rod-shaped skeleton structure [26]. However, with the further conservation of the cavitating jet treatment time, the mesh skeleton structure was seriously broken and gradually transformed into a short bar structure. When the treatment time reached 15 min, the mesh structure disappeared, and the skeleton structure presented a slender bar. Combined with the above results, the cavitation jet has the dual effects of breaking and reassembling the protein skeleton structure. In addition, it also induces a mutual transformation between the soluble and insoluble aggregates. Therefore, the long-time cavitation jet can induce the soluble aggregates to transform into insoluble aggregates and be removed by centrifugation, and decrease the content of the soluble aggregates. In addition, under high temperatures, great pressure, and the shear force conditions of the long-time cavitation jet, the skeleton structure of the protein was broken. These two works together resulted in the decrease of the cementation and intercross network structure of the soluble aggregates, and the formation of a small and slender skeleton structure.

### 3.6. Emulsion Capacity and Stability

Due to its good emulsifying activity, proteins are usually used in food emulsions and artificial fats. However, emulsifying features differ both on the capability of the protein adsorbed on the oil droplet superficially and the protein intermolecular binding, and it is related to the shape, size, and superficial hydrophobicity of the protein molecules [45]. The EAI and ESI of the emulsions stabilized by the soluble soybean protein oxidized accumulates are shown in Table 4. The oxidization treatment decreased the EAI and ESI of emulsions steadied by OSPI and SOSPI. In addition, the EAI of SOSPI was superior to that of SPI, but the ESI was relatively inferior. The oxidation treatment could form a highly ordered intermolecular β-sheet between proteins, which were poor supports to the flexible body. The molecular flexibility of OSPI decreased and formed insoluble oxidized aggregates whose structure is difficult to relax, resulting in the reduction of protein interface activity and the decline of the binding ability between the protein and oil, which induced the EAI and ESI of OSPI, inferior than that of SPI [46]. However, compared with the OSPI, the SOSPI contained abundant free sulfhydryl groups and short worm-like skeleton structures. In the process of forming the emulsions, the SOSPI moved to the interface in smaller particle size aggregates, which increased the exchange area between the protein and the oil–water interface and made SOSPI easier to absorb and relax at the interface, so that the EAI of SOSPI were higher than that of OSPI. Nevertheless, it is difficult for proteins with a miniature particle size to adsorb stably on the interface for a long time, resulting in a decrease of the ESI of SOSPI.

With the extension of the cavitation jet treatment time, the EAI and ESI of SOSPI initially amplified and then declined, and achieved the highest when the cavitation jet treatment time was 6 min. The high pressure, shear, and cavitation effects shaped by the cavitating jet could cleave oxidized aggregates and induce some insoluble aggregates to change into soluble aggregates, so that the content of the soluble protein accumulates increased. The cavitation jet could also abolish the intermolecular binding of SCOSPI, and the structure of SCOSPI is altered. These two results result in the amplification of the number of exterior hydrophobic parties and polar groups, particle size, and molecular flexibility of SCOSPI to improve the emulsifying activity and emulsifying constancy [47]. Additionally, the intercross networks structure, cross-connected by the protein, were useful in the creation of the emulsion [48]. Combined with the TEM results, it can be found that after the cavitation jet treatment, the mesh skeleton structure of SCOSPI became larger than was beneficial to the formation and stability of the emulsion. However, when the cavitation jet treating time exceeded 6 min, the long-time high temperature, pressure, and shear force produced by cavitation jet impacted the hydrophilic and hydrophobic clusters and interior binding of the protein molecules, which unfavored the aptitude of SCOSPI to adsorb at the oil–water interface. In addition, during the long time cavitation jet, the SCOSPI gradually formed a small molecular protein with a more highly ordered β-sheet structure, difficult relaxation, and low molecular flexibility, resulting in the reduction of the emulsifying activity and emulsifying stability of SCOSPI [49].

### 3.7. Confocal Laser Scanning Microscope (CLSM)

**Figure 6:**
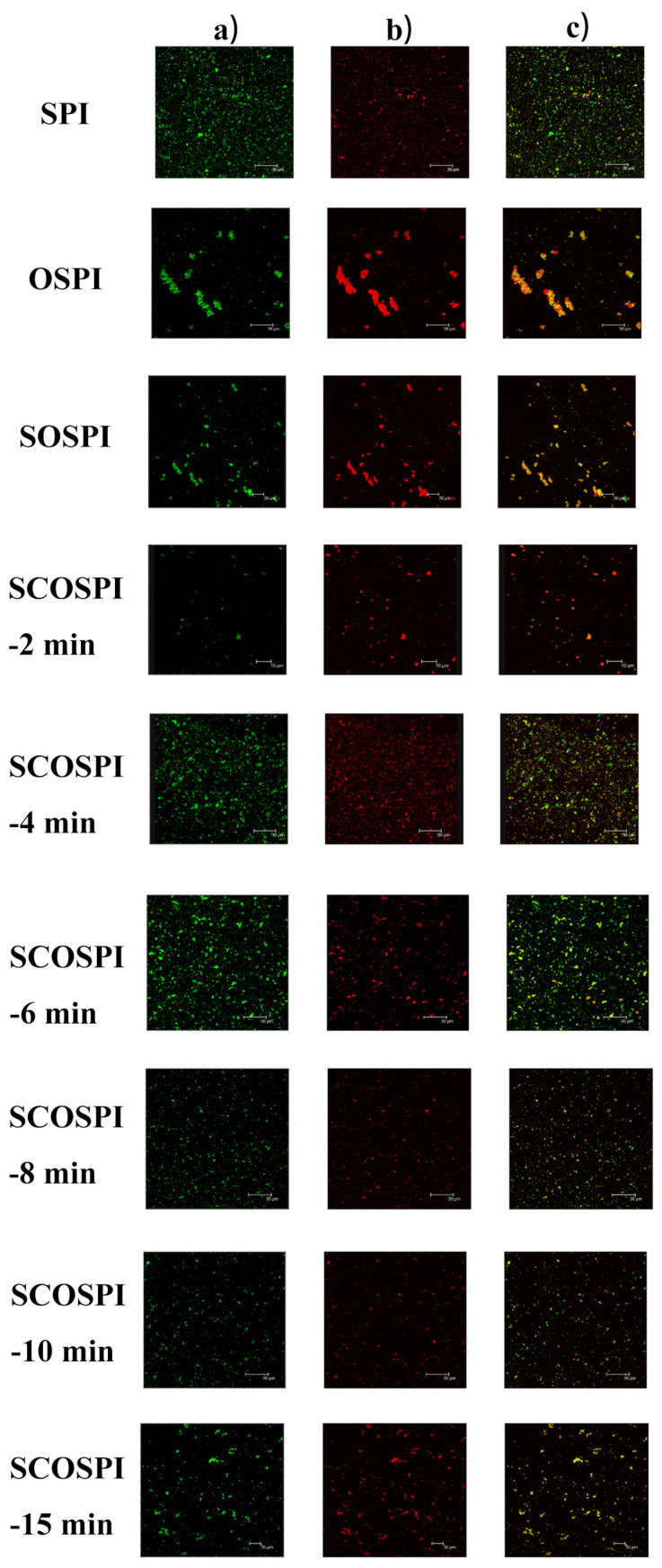
CLSM micrographs of natural, oxidized soybean protein, and cavitation jet treated on soluble soybean protein oxidized accumulates at several times (2, 4, 6, 8, 10, and 15 min). Note: (**a**) represents the proteins, (**b**) represents the oil droplets, and (**c**) represents the proteins adsorbed on the oil droplet.

The CLSM was utilized to explore the fluctuations in the microstructure of the soybean protein emulsion after pretreating it with the cavitation jet, as displayed in Figure 6. The green fluorescence in CLSM micrographs signified the protein piece, the red fluorescence represented the soybean oil, and the vivid yellow fluorescence signified the proteins adsorbed on the oil droplets. The small spherical droplets were evenly distributed throughout the emulsion system, which was prepared from SPI. After the oxidation treating, the flocculation of the emulsion dewdrops prepared from OSPI and SOSPI were serious, and the red areas in the unceasing phase increased significantly and gathered on the surface of emulsion droplets, showing the phenomenon of oil–water separation. The oil–water separation of OSPI was more serious than SOSPI. The oxidative treatment caused the accumulation and solubility of the protein molecules to decrease, causing difficulty for the formation of steady interfacial film in the emulsification procedure, thus showing a red oil droplet accretion area in a large aggregation area [50]. Nevertheless, compared with OSPI, the particle size and skeleton structure of SOSPI were smaller, and it had better adsorption ability at the oil–water interface. Most of the oil droplets are encapsulated in the emulsion droplets prepared by SCOSPI, and there was only a small-scale accumulation of red oil droplets. This shows that macromolecules and insoluble protein oxidation aggregates formed during the oxidation were the key to causing significant decline in emulsifying activity. Therefore, regulating the macromolecules and insoluble proteins aggregates could improve the function activity of OSPI.

During the treatment of the cavitation jet, the green area of the emulsion prepared with SCOSPI in the CLSM image increased gradually, and the red area gradually decreased and showed the wrapped state in the green area, and a steady protein interfacial film was shaped at the oil–water interface. The cavitation jet treatment could induce the insoluble oxidation aggregates, which are difficult to relax at the interface, to transform into soluble aggregates with smaller steric hindrance and a more flexible structure, as well as to expand the adsorption, relaxation, and reordering effects of SCOSPI at the interface, so as to improve the steadiness of the oil–water interface [51]. In addition, the number of surface hydrophobic groups and polar groups in SCOSPI were increased, which promoted additional protein molecules to be adsorbed at the oil–water interface to produce a steadier interfacial film, and could improve the interfacial activity and emulsifying features [52,53]. Therefore, the proteins discolored green were consistently and firmly spread across the oil droplets that efficiently avoided coalescence. However, with the further delay of the cavitation jet treatment time, in the CLSM images of the emulsion prepared by SCOSPI, the red area of oil increased gradually, and the green area of the protein was mainly concentrated. Excessive cavitation jet treatment would increase the content of the anti-parallel intermolecular β-sheet, surface hydrophobicity, and ζ-potential reduction of SCOSPI, and the specific surface area declined, which was not encouraging for distribution to crossing and extension on the oil–water interface [54]. Finally, the binding capacities of SCOSPI to oil were weakened, resulting in the increase of free oil droplets and serious oil–water separation. A cavitation jet can affect the flocculation and stability of the emulsion by regulating the transformation between insoluble and soluble aggregates.

### 3.8. Quantity of Adsorbed Proteins at Interface (AP%)

**Figure 7:**
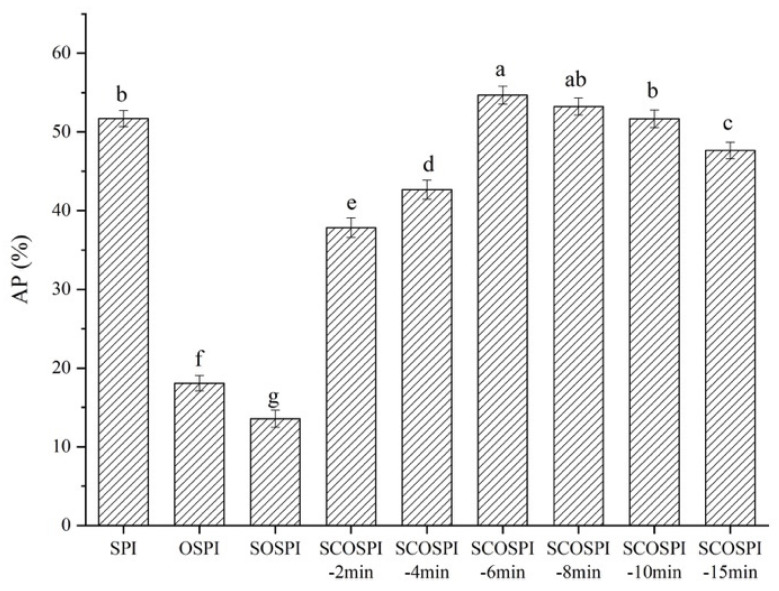
AP% of emulsions of natural, oxidized soybean protein, and cavitation jet treated on soluble soybean protein oxidized accumulates at several times (2, 4, 6, 8, 10, and 15 min). Note: Values with a different letter(s) indicate a significant difference at *p* ≤ 0.05.

The quantity of the adsorbed proteins at the interface has a key impact on the constancy of the emulsion. The higher interfacial protein amount, the stronger capability of protein adsorption to the oil–water interface [55]. As shown in Figure 7, the AP% of the emulsion made by OSPI and SOSPI decreased compared with SPI, and the AP% of the emulsion made by SOSPI was lower than OSPI. It is generally considered that proteins undergo a certain amount of structural extension and relaxation when dissolved in an aqueous solution [56]. The more flexible the structure of the proteins, the better it is for structural expansion and the more prone they are to bulk diffusion, adsorption at the interface, unfolding, and rearrangement, inducing the increase of AP% [57]. During oxidation, the level of protein accumulation creased, and the structural flexibility of highly clustered OSPI was reduced and its rigidity was enhanced, which was not favorable to the expansion and adsorption at the interface, resulting in the decrease of AP% [58]. In addition, the decrease of AP% might be largely ascribed to the fabrication of the bridged emulsions, *e.g.,* two particular oil droplets allocated an identical protein particle layer, facilitated by the oxidization-induced aggregation of SPI [59]. At the time of the formulation of the emulsion, large molecules can be transported to the oil–water interface in preference to small molecules because of the convective mass transport effect of high-pressure homogenization, i.e., large molecules can adsorb more quickly than small molecules [60]. Compared with OSPI, SOSPI had a small particle size and low molecular weight, which affected the protein adsorption ratio to the oil–water interface, subsequent in lower AP% [61]. However, the protein with a small particle size, low molecular weight, and a high solubility contributes to improving the connection region with the oil–water interface and the affinity of protein to the interface; foremost there was only a small-scale accumulation of red oil droplets in the emulsion prepared by SOSPI. Therefore, the AP% is not the only consideration in deciding the characteristics of emulsion.

With the expansion of the cavitation jet treatment time, the AP% of SCOSPI boosted first and then diminished, and touched the highest when the treatment time was 6 min. The high-velocity turbulent flow, high-speed shearing, and large pressure produced by the cavitation jet acted on cross-linked aggregates to realize the transformation from insoluble aggregates to soluble aggregates and directional regulation for amorphous soluble aggregation, which improved the soluble protein content and molecular flexibility of the soluble proteins. It promoted more proteins with structural relaxation and overall flexibility to be adsorbed, unfolded, and rearranged at the interface. Then, it enhanced the AP% of the emulsion prepared by SCOSPI, and increased to interfacially prepare and bulk stabilize the oil–water systems [62]. Nevertheless, when the cavitation jet treating time was too long, the majority of the soluble protein components were dominated by a small molecular weight and particle size protein molecules, and the content of β1 in SCOSPI was increased, designating that the content of the methodical structure augmented and that the structure was relatively tight and complex, which was not favorable for the adsorption and unfolding of protein at the oil–water interface and the formation of a dense interface interfacial film. These results together led to the decrease of AP% of emulsion prepared by SCOSPI [63].

### 3.9. Interfacial Tension

**Figure 8:**
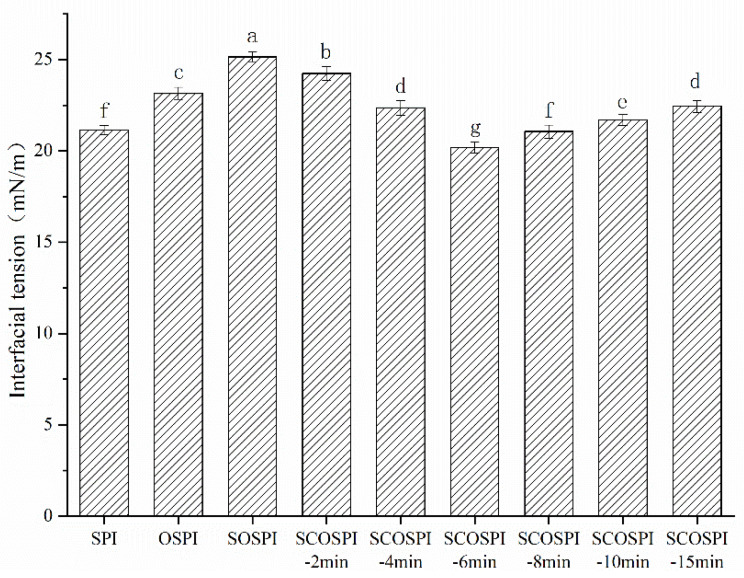
Interfacial tension of emulsions of natural, oxidized soybean protein, and cavitation jet treated on soluble soybean protein oxidized accumulates at several times (2, 4, 6, 8, 10, and 15 min). Note: Values with a different letter(s) indicate a significant difference at *p* ≤ 0.05.

A key element in the investigation and analysis of emulsion stability is the interfacial tension at the liquid–liquid interface, which can also describe the exterior activity of proteins at the oil–water interface [64]. As shown in Figure 8, the interfacial tension of the emulsion prepared with natural soy protein was 21.29 mN/m. After the oxidation treatment, the interfacial tension of the emulsions made by OSPI and SOSPI were raised, and SOSPI was higher than OSPI. The oxidation treatment would promote a protein aggregate to form insoluble oxidation aggregates with a larger particle size, low molecular flexibility, and poor solubility [39]. In addition, the exact surface region of the protein molecules was reduced, and the steric hindrance was increased, which were not beneficial to the adsorption and reordering of protein at the oil–water interface, resulting in an increase in the protein interfacial tension [65]. The soluble components in SOSPI were more easily dissolved in the water phase, the particle size was lesser, and the ordered structure was greater. The ability to form stable interfacial film was significantly reduced, resulting in a higher interfacial tension and lower emulsion stability.

After the cavitation jet treatment, the interfacial tension of SCOSPI decreased first and was then amplified, achieving the bottom when the treatment time was 6 min. Combined with the results of the particle distribution and TEM, it could be seen that the particle size of SCOSPI increased and the protein skeleton widened, indicating that the cavitation jet could break the insoluble soybean protein oxidation aggregate and transform it into soluble oxidation aggregate, resulting in the increase of hydrophobic clusters of soluble protein components and a more complex conformational space [53]. Protein molecules were not easily soluble in the aqueous phase, which improved the expansion and reordering of the protein molecules at the interface and declined the interfacial tension [66]. However, when the cavitation jet treatment time was too long, the quantity of β1 increased and the γ-random coil (Table 2) decreased. Additionally, particle size (Table 1) was reduced and soluble oxidized aggregates with a more orderly structure and lower molecular flexibility were formed, with the result that adsorption energy barricaded at the boundary was higher, and the adsorption efficacy was decreased. This affected the adsorption and evolving of the protein at the oil–water interface and caused an increase of interfacial tension [67]. Combined with the results of EAI, ESI, CLSM, and AP%, we can find that when the cavitation jet treatment was 6 min, the soluble soybean protein oxidized aggregates showed the best emulsification interface characteristics. This provided a simple and effective technology for the application of soybean protein in the food industry.

### 3.10. Viscoelastic Properties

**Figure 9:**
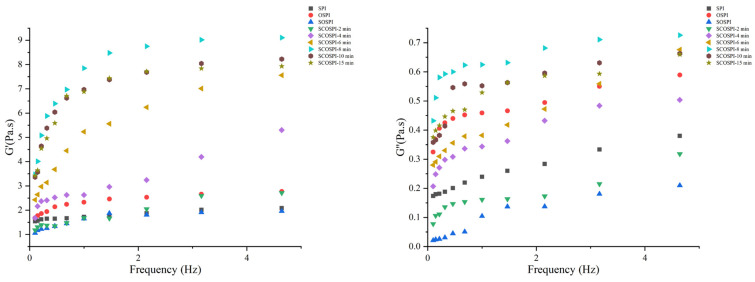
Viscoelastic properties of natural, oxidized soybean protein, and cavitation jet treated on soluble soybean protein oxidized accumulates at several times (2, 4, 6, 8, 10, and 15 min).

Rheological quantities deliver evidence on the physical performance and steadiness of lotion [68]. Elastic modulus G′ is a measurement of elasticity and signifies the storing modulus of the energy of stress that could be reinstated when the stress is liberated, while the viscous modulus G″ signifies the viscous substances, which assumes the flow defiance of the sample [69,70]. G’ and G″ of emulsion are shown in Figure 9. Both the elastic modulus (G′) and viscous modulus (G″) gradually amplified within the oscillation frequency range. In all samples, the G′ was superior to the G″ and exhibited an elastic character. It showed that the protein on the interface formed a viscoelastic adsorbed film and suggested an elastic network structure of emulsion. The G′ and G″ of the emulsion formulated by OSPI were both higher than SPI, while the SOSPI showed the opposite results. The rheological features of the interface layer were mainly impacted by the hydrophobic interaction and disulfide bonds among proteins adsorbed at the oil–water interface [71]. After the oxidation treatment, the quantity of the disulfide bonds in the protein aggregates increased significantly. In addition, under the hydrophobic interaction, they were bound to the proteins that have been adsorbed on the interface layer. Therefore, the formation of the oxidative aggregates enhanced the binding between protein molecules at the interface, thus amending the interfacial elastic modulus of the emulsion prepared by OSPI. However, the soluble soybean protein aggregates were transformed into insoluble oxidized aggregates after the oxidation treatment. The soluble components with smaller molecular proteins were more likely to dissolve in the water phase and were unable to shape a protein-bound film, resulting in a decline in G′ and G″ of emulsion equipped by SOSPI [72].

With the cavitation jet treatment, the G’ of the emulsion fabricated by SCOSPI increased first and then declined, and the G″ presented no obvious change. With the delay of cavitation jet treatment time, the skeleton of soluble oxidized aggregates became wider, the particle size was larger, the exposure of hydrophilic and lipophilic groups in components raised, the electrostatic revulsion among emulsion droplets was also amplified, the protein content adsorbed on the oil–water interface layer was more and more, and the thickness of the interfacial film slowly augmented, subsequent to the increase of interfacial elastic modulus. When the cavitation jet treating time was maximized, the SCOSPI through β1 would form protein aggregates with a high aggregation degree, small particle size, and weak reticular structure, which had negative impacts on the interface activity and resulted in the decrease of G’ [73,74].

### 3.11. Apparent Viscosity

**Figure 10:**
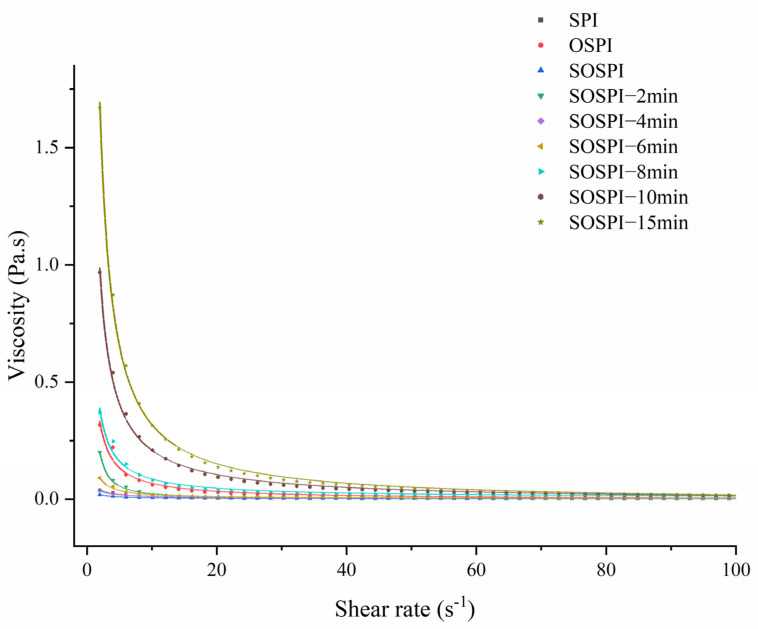
Apparent viscosity and shear rate relationship of emulsions of natural, oxidized soybean protein, and cavitation jet treated on soluble soybean protein oxidized accumulates at several times (2, 4, 6, 8, 10, and 15 min).

Figure 10 and Table 5 depict the emulsions’ rheological behavior. All the emulsions’ flow curves could be matched with Sisko’s model. With flow performance indices fluctuating from 0.059 to 0.271, all the emulsions displayed shear-thinning behavior. Intermolecular bindings among aggregated molecules, which result in the creation of weak transient networks, may be the motive of shear-thinning actions for the stabilized emulsions [65]. Emulsions steadied by OSPI exhibited a higher ostensible viscosity and K than those stabilized by SPI, but those steadied by SOSPI had an inverse relationship between their apparent viscosity and K. The volume proportion of the dispersed phase and the size of the combinations created from the proteins determined the rheological parameters of the emulsion. The higher the number and size of these masses, the higher the viscosity [75,76]. This led to a higher initial apparent viscosity in the emulsion made from oxidized soy protein aggregates. After the oxidation process, the initially soluble oxidized aggregates eventually changed into insoluble oxidized aggregates. A reduction in the K and apparent viscosity resulted from the remaining soluble components, which were primarily made up of tiny protein molecules that tended to dissolve in the aqueous phase and enclose oil droplets [42,77,78]. The ostensible viscosity and K of the emulsion produced by SCOSPI grew initially and subsequently dropped with the length of the cavitation jet treatment time. Particle size and TEM results show that an effective cavitation jet treatment could encourage the conversion of insoluble aggregates into soluble aggregates, increase the particle size and skeleton structure of soluble soybean protein oxidation masses, and ultimately, increase the ostensible viscosity and K of the emulsion created by SCOSPI. However, the prolonged cavitation jet treating period might lead to an increase in insoluble aggregates that were unfavorable to the steadiness of the emulsion, lowering K and its apparent viscosity. By modifying the structure and content of the soy protein soluble oxidized accumulates and controlling the reciprocal transformation of protein constituents, the cavitation jet treatment can alter the rheological characteristics of the emulsion.

## 4. Conclusions

Oxidized treatment influenced the structure of the SOSPI, causing a decline in their emulsifying properties and interfacial features. A cavitation jet at a short treating time can break the insoluble soybean protein oxidation aggregate and transform it into a soluble oxidation aggregate, causing the expansion of particle size, protein skeleton, and disulfide bond content. This also improved the emulsion activity and state of SOSPI and raised the quantity of adsorbed proteins at the interface while decreasing the interfacial tension of the emulsion. A long cavitation jet treatment time could induce the soluble oxidized aggregate to gradually form a small molecular weight protein with difficult relaxation and low molecular flexibility, which were not favorable to the solidity of emulsion, resulting in the decrease of EAI, ESI, apparent viscosity, K, and an increase of interfacial tension.

## Figures and Tables

**Table 1:** Particle size and protein dispersibility index (PDI) of natural, oxidized soybean protein, and cavitation jet treated on soluble soybean protein oxidized accumulates at several times (2, 4, 6, 8, 10, and 15 min).

Samples	Particle Size (nm)	PDI
SPI	181.59 ± 2.77 ^h^	0.76 ± 0.11 ^a^
OSPI	3836.18 ± 66.82 ^a^	0.19 ± 0.03 ^d^
SOSPI	97.38 ± 1.63 ^i^	0.17 ± 0.01 ^d^
SCOSPI-2 min	551.03 ± 5.07 ^e^	0.14 ± 0.02 ^ef^
SCOSPI-4 min	639.27 ± 18.31 ^d^	0.11 ± 0.04 ^f^
SCOSPI-6 min	705.81 ± 15.33 ^c^	0.29 ± 0.04 ^c^
SCOSPI-8 min	776.14 ± 11.91 ^b^	0.15 ± 0.01 ^e^
SCOSPI-10 min	538.15 ± 9.52 ^f^	0.28 ± 0.03 ^c^
SCOSPI-15 min	443.96 ± 22.28 ^g^	0.33 ± 0.01 ^b^

Note: Comparisons were carried out between values of the same column; values with a different letter(s) indicate a significant difference at *p* ≤ 0.05.

**Table 2:** Secondary structure content of natural, oxidized soybean protein, and cavitation jet treated on soluble soybean protein oxidized accumulates at several times (2, 4, 6, 8, 10, and 15 min).

Content (%)	Anti-Parallel Intermolecular β-Sheet (β1)	Parallel Intermolecular β-Sheet (β2)	Intramolecular β-Sheet	ɑ-Helix	β-Turn	γ-Random Coil
Wavenumber (cm^−1^)	1608–1622	1682–1700	1622–1637	1646–1662	1662–1681	1637–1645
SPI	9.27 ± 0.28 ^d^	15.95 ± 0.23 ^a^	18.41 ± 0.18 ^a^	26.35 ± 0.18 ^c^	21.33 ± 0.17 ^f^	8.69 ± 0.15 ^e^
OSPI	13.58 ± 0.19 ^a^	7.16 ± 0.18 ^f^	17.18 ± 0.13 ^b^	17.82 ± 0.19 ^d^	26.66 ± 0.20 ^e^	17.41 ± 0.20 ^a^
SOSPI	9.05 ± 0.26 ^d^	9.74 ± 0.22 ^b^	13.58 ± 0.25 ^f^	27.84 ± 0.19 ^a^	28.28 ± 0.18 ^d^	10.51 ± 0.22 ^b^
SCOSPI-2 min	11.28 ± 0.08 ^c^	8.54 ± 0.26 ^d^	13.38 ± 0.07 ^g^	26.27 ± 0.08 ^c^	30.61 ± 0.13 ^b^	9.92 ± 0.17 ^c^
SCOSPI-4 min	12.70 ± 0.10 ^b^	7.84 ± 0.19 ^e^	13.80 ± 0.26 ^e^	24.52 ± 0.19 ^e^	31.82 ± 0.02 ^a^	9.32 ± 0.18 ^d^
SCOSPI-6 min	12.77 ± 0.01 ^b^	9.02 ± 0.25 ^c^	13.58 ± 0.05 ^f^	23.36 ± 0.04 ^f^	31.92 ± 0.22 ^a^	9.35 ± 0.06 ^d^
SCOSPI-8 min	11.42 ± 0.19 ^c^	9.83 ± 0.01 ^b^	15.80 ± 0.19 ^c^	22.72 ± 0.14 ^g^	29.66 ± 0.22 ^c^	10.57 ± 0.16 ^b^
SCOSPI-10 min	12.78 ± 0.06 ^b^	6.78 ± 0.22 ^g^	13.70 ± 0.03 ^ef^	26.68 ± 0.20 ^b^	30.73 ± 0.24 ^b^	9.33 ± 0.30 ^d^
SCOSPI-15 min	13.51 ± 0.13 ^a^	6.76 ± 0.05 ^g^	15.36 ± 0.15 ^d^	25.09 ± 0.06 ^d^	29.79 ± 0.15 ^c^	9.49 ± 0.18 ^d^

Note: Comparisons were carried out between values of the same column; values with a different letter(s) indicate a significant difference at *p* ≤ 0.05.

**Table 3:** Sulfhydryl content of natural, oxidized soybean protein, and cavitation jet treated on soluble soybean protein oxidized accumulates at several times (2, 4, 6, 8, 10, and 15 min).

Samples	Free Sulfhydryl (nmol/mg)	Total Sulfhydryl (nmol/mg)	Disulfide Bond (nmol mg^−1^)
SPI	11.72 ± 0.15 ^a^	15.64 ± 0.32 ^a^	1.96 ± 0.11 ^c^
OSPI	4.05 ± 0.23 ^h^	10.78 ± 0.14 ^g^	3.37 ± 0.13 ^a^
SOSPI	8.06 ± 0.22 ^e^	10.99 ± 0.12 ^f^	1.47 ± 0.16 ^d^
SCOSPI-2 min	8.65 ± 0.17 ^c^	11.95 ± 0.16 ^e^	1.65 ± 0.17 ^cd^
SCOSPI-4 min	8.92 ± 0.21 ^b^	12.56 ± 0.18 ^c^	1.82 ± 0.13 ^c^
SCOSPI-6 min	8.87 ± 0.19 ^bc^	13.29 ± 0.22 ^b^	2.21 ± 0.15 ^b^
SCOSPI-8 min	8.21 ± 0.14 ^d^	12.15 ± 0.19 ^d^	1.97 ± 0.16 ^c^
SCOSPI-10 min	7.65 ± 0.16 ^f^	10.69 ± 0.15 ^g^	1.52 ± 0.16 ^d^
SCOSPI-15 min	7.15 ± 0.18 ^g^	10.07 ± 0.16 ^h^	1.46 ± 0.19 ^d^

Note: Comparisons were carried out between values of the same column; values with a different letter(s) indicate a significant difference at *p* ≤ 0.05.

**Table 4:** Emulsion capacity and solidity of natural, oxidized soybean protein, and cavitation jet treated on soluble soybean protein oxidized accumulates at several times (2, 4, 6, 8, 10, and 15 min).

Samples	EAI/(m^2^·g^−1^)	ESI/min
SPI	91.61 ± 1.17 ^a^	186.19 ± 3.89 ^a^
OSPI	46.97 ± 2.24 ^g^	137.20 ± 3.51 ^e^
SOSPI	89.92 ± 2.37 ^a^	126.75 ± 3.18 ^f^
SCOSPI-f-2 min	57.17 ± 1.89 ^f^	149.08 ± 3.15 ^d^
SCOSPI-f-4 min	71.92 ± 1.91 ^e^	156.24 ± 2.89 ^c^
SCOSPI-f-6 min	86.24 ± 2.39 ^b^	163.17 ± 2.68 ^b^
SCOSPI-f-8 min	81.64 ± 2.64 ^c^	152.56 ± 3.37 ^cd^
SCOSPI-f-10 min	76.95 ± 2.19 ^d^	147.89 ± 2.90 ^d^
SCOSPI-f-15 min	72.68 ± 2.64 ^e^	140.27 ± 3.64 ^e^

Note: Comparisons were carried out between values of the same column; values with a different letter(s) indicate a significant difference at *p* ≤ 0.05.

**Table 5:** Fitting result of Sisko’s model of emulsion of natural, oxidized soybean protein, and cavitation jet treated on soluble soybean protein oxidized accumulates at several times (2, 4, 6, 8, 10, and 15 min).

Samples	*K* (Pa·s^n^)	*n*	R^2^
SPI	0.066	0.271	0.99
OSPI	0.626	0.059	0.99
SOSPI	0.021	0.169	0.99
SCOSPI-2 min	0.539	0.183	0.99
SCOSPI-4 min	1.380	0.216	0.99
SCOSPI-6 min	2.673	0.219	0.99
SCOSPI-8 min	3.343	0.173	0.99
SCOSPI-10 min	3.123	0.154	0.99
SCOSPI-15 min	2.676	0.152	0.99

Note: The *K* signifies the consistency index. The *n* signifies the behavior index.

## Data Availability

The data presented in this study are available on request from the corresponding author.
